# Extraction and characterization of collagen from the skin of Amazonian freshwater fish pirarucu

**DOI:** 10.1590/1414-431X2023e12564

**Published:** 2023-05-15

**Authors:** K.C.R. Carpio, R.S. Bezerra, T.B. Cahú, F.T.D. do Monte, R.C.A. Neri, J.F. da Silva, P.R. dos Santos, R.P. Carvalho, D.M.L. Galeno, A.J. Inhamuns

**Affiliations:** 1 Programa de Pós-graduação em Biotecnologia, Departamento de Fisiologia, Instituto de Ciências Biológicas, Universidade Federal do Amazonas, Manaus, AM, Brasil; 2 Laboratório de Enzimologia, Departamento de Bioquímica, Universidade Federal de Pernambuco, Recife, PE, Brasil; 3 Laboratório de Tecnologia do Pescado, Faculdade de Ciências Agrárias, Universidade Federal do Amazonas, Manaus, AM, Brasil; 4 Departamento de Fisiologia e Comportamento, Universidade Federal do Rio Grande do Norte, Natal, RN, Brasil

**Keywords:** Arapaima gigas, Biomolecules, By-product, Sustainability, Collagen extraction

## Abstract

The need to fully exploit fishing resources due to increasing production and consequent waste generation requires research to promote the sustainability of the fishing industry. Fish waste from the industry is responsible for relevant environmental contamination. However, these raw materials contain high amounts of collagen and other biomolecules, being attractive due to their industrial and biotechnological applicability. Thus, to reduce the waste from pirarucu (*Arapaima gigas*) processing, this study aimed to obtain collagen from pirarucu skin tissue. The extraction process used 0.05 M sodium hydroxide, 10% butyl alcohol, and 0.5 M acetic acid, with extraction temperature of 20°C. The obtained yield was 27.8%, and through sodium dodecyl sulfate polyacrylamide gel electrophoresis (SDS-PAGE), it was determined that the collagen obtained was type I. This study showed that collagen solubility was highest at pH 3 and the lowest solubility was at concentrations of 3% sodium chloride. The denaturation temperature of collagen was 38.1°C, and its intact molecular structure was observed using the Fourier transform infrared spectrophotometry technique with an absorption radius of 1. The results showed that it was possible to obtain collagen from pirarucu skin at 20°C, which has the typical characteristics of commercial type I collagen. In conclusion, the procedures used may be considered to be an interesting alternative for collagen extraction, a new product obtained from the processing of fish waste.

## Introduction

Collagen is present in all multicellular animals, providing mechanical support, resistance, and elasticity to several organs and tissues (1). Collagen is the most abundant extracellular matrix protein and represents one third of all proteins in humans. It is the main component of connective tissue, being related to metabolic and genetic disorders such as scurvy, osteogenesis imperfecta, and Ehlers-Danlos syndrome (2).

Collagen is considered one of the most used biomaterials for industrial applications, and because of its benefits, many researchers study alternative sources of this protein, especially from aquatic organisms (3). There are several procedures for extracting this protein from aquatic organisms. However, the best conditions depend on the thermal stability of the biomolecule, which is different in fish from temperate and tropical regions. This is important to obtain a better yield, purity, and integrity of the collagen molecular structure (4).

Collagen from fishing residues has been applied in the cosmetic, food, biomedical, and pharmaceutical industries in the form of supplements, additives, film formation, and edible coatings because of its functional properties and protein content (5). It can be successfully used in the biomedical industry as a regenerator of skin and bone tissue and in the healing of wounds, blood vessels, and ligaments (6). In the cosmetics industry, collagen is widely used in the prevention and reduction of wrinkles, as masks and skin injections, in the prevention of hair loss and hair weakness, and to improve the appearance of the skin.

For decades, the processing of different fish species has generated many waste products, causing economic losses and environment contamination. However, depending on the source, these may be considered as trash or delicacy (4).

International organizations such as the Food and Agriculture Organization of the United Nations (FAO) and the Common Fisheries Policy (CFP) of the European Union are focused on reducing waste from the fishing production process, which disposes of up to 70 million tons annually into the environment (7). Because of this, measures are being taken that contribute to the protection of our planet, such as the use of these residues for the extraction of biomolecules of industrial interest with biotechnological application and for the generation of bioeconomic capital (8).

The *Arapaima gigas*, known as pirarucu in Brazil, is an important source of protein, especially in Amazonian culture. The by-products and/or residues, skins, and scales, equally rich in protein, are improperly discarded in rivers, impacting the ecosystem (9). It is cultivated in sustainable development reserves (RDS) and in Brazilian fish farms, with an estimated production of 172,599.81 tons, being the main income of most producers and riverside communities involved in this activity (10).

Most fish residues are from smaller species that nevertheless give excellent results in biomolecules extraction. However, medium and large species such as pirarucu have been of greater commercial and technological interest in recent years, as they have losses or residues from their processing of up to 56% of the fillet weight, making it attractive for the extraction of biomolecule of industrial interest, such as collagen (11,12).

The production of collagen from the skin obtained from the processing of the pirarucu encourages the elaboration of protocols for the full use of these fishing residues, making this production increasingly attractive as the skin would not be wasted, showing new ways of creating a bioeconomy in this production chain. Thus, to reduce the waste from pirarucu processing, this study aimed to extract collagen from the pirarucu skin.

## Material and Methods

**Figure 1:**
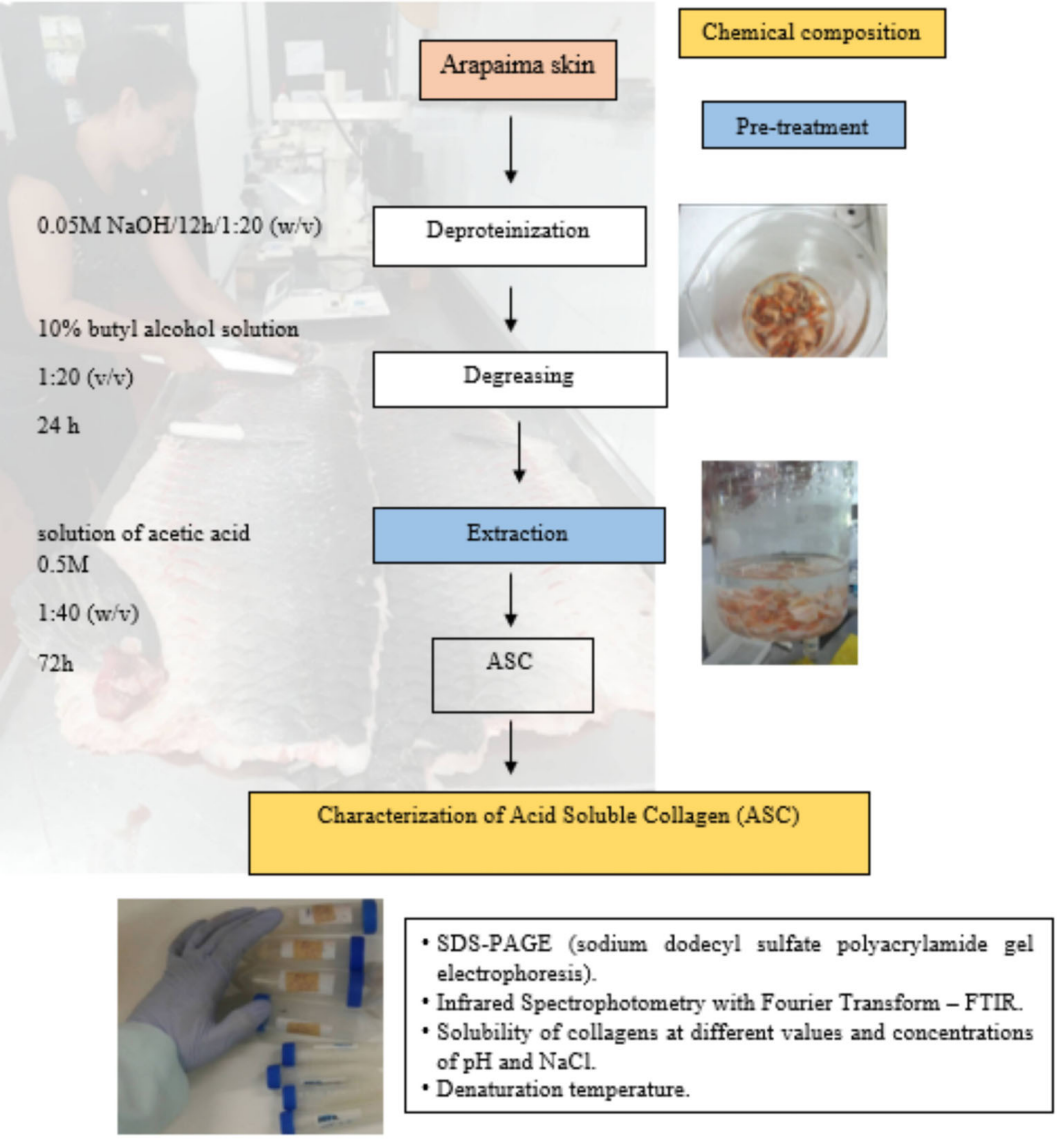
Experimental protocol.

Type I collagen from steer skin was obtained from Sigma (USA) and markers of high molecular weight were obtained from GE Healthcare UK Limited (UK) for evaluation of protein molecular weight. All chemical reagents were of analytical grade. The experimental protocol is shown in Figure 1.

### Collection and preparation of skin tissue

The pirarucu skin tissue, from a commercial production system, was obtained frozen from Kardume Fish Trade Ltd. (Brazil) and transported to the Fish Technology Laboratory of Federal University of Amazonas. The skin was washed with ice-cold distilled water, cut into approximately 1.0-cm^2^ pieces, and stored in a freezer at -20°C until use. Analyses of centesimal composition were performed in triplicate.

### Extraction process from pirarucu skin tissue

The acid-soluble collagen (ASC) extraction process was performed as previously described with some modifications (4). A working temperature of 20°C was maintained with constant homogenization in an ultra-thermostatic bath with water circulation (QUIMIS model Q214M, Brazil) linked to a mechanical stirrer (NOVA model NI 1133, Brazil) until collagen was obtained.

The extraction process was developed in two main stages. The first step used a 0.05 M NaOH solution for 12 h, exchanging solution every 6 h, in a ratio of 1:20 (w/v). Then, the samples were rinsed with ice-cold distilled water until neutral pH. The samples were subjected to a 10% butyl alcohol solution at a ratio of 1:20 (v/v) for 24 h, with solution changes every 12 h, and then rinsed with distilled water at 4°C, preparing the solution for the extraction of collagen.

In the second stage, a solution of acetic acid was used at a concentration of 0.5 M and proportion of 1:40 (w/v) for 72 h, with changes every 24 h. Once the viscous solution was obtained, buffering was performed with a 0.9 M carbonate-bicarbonate buffer solution and 0.9 M sodium chloride until a pH of 8.87.

Thereafter, centrifugation was done at 4°C at 11,000 *g* for 40 min. The material collected from the centrifugation was submitted to three dialysis processes. The first in a solution of 0.1 M acetic acid and the last two dialyses in ice-cold distilled water. Each dialysis was performed for 12 h. The dialyzed material was frozen to be lyophilized to obtain the dried ASC.

The extraction yield was calculated on dry matter basis according to the formula (13): *Yield (%)* = (*M . M*_0_^−1^) × 100, where M is the dry weight (g) of lyophilized material and M_0_ is the weight of dry skin tissue used (g).

### Characterization of ASC

For the characterization of the lyophilized material as type I collagen, SDS-PAGE (sodium dodecyl sulfate polyacrylamide gel electrophoresis) was performed using 4% (w/v) stacking gel and 12.5% separation gel (14). The sample of lyophilized material was dissolved in 1% (w/v) SDS at a concentration of 2 mg/mL, and the sample buffer was 0.5 M Tris-HCl, pH 6.8, containing SDS 4% (w/v), 20% glycerol (v/v) with 10% (v/v) beta-mercaptoethanol in a ratio of 1:4 (v/v).

Then, 25 μL aliquots of the samples were heated at 100°C for 10 min in a bath (IKA^®^ Works 144 Inc., China), placed on polyacrylamide gel, and subjected to vertical electrophoresis (15 mA gel^-1^) (Electrophoresis System Bio-Rad Laboratories, Inc., China). The gel was stained with Coomassie blue 0.05% (w/v) in 15% methanol (v/v) and 5% (v/v) acetic acid for 10 min and decolorized with a solution of 30% (v/v) methanol and 10% (v/v) acetic acid for 12 h. Bovine type I collagen (Sigma-Aldrich, USA) was prepared following similar procedures of samples, and 10 μL of it was used as standard collagen. High molecular weight markers (GE Healthcare UK Limited) were used to estimate the molecular weight of proteins.

The ASC sample was also analyzed by Fourier-transform infrared spectrophotometry (FTIR) (Cary 630 Agilent Technologies, USA). The signals were collected automatically in a range of 4000-400 cm^-1^, at a resolution of 2 cm^-1^, and compared to a background spectrum collected from the clean empty cell.

The collagen solubility was assessed according to a previously published protocol with some adaptations (15). The final concentration was 3 mg/mL in 0.5 M acetic acid solution. Different pH-adjusted (1.0-12.0) concentrations were prepared separately with 6 M NaOH and 6 M HCl (hydrochloric acid) solutions. Each ASC sample was homogenized in each solution of different pH values (v/v) and subsequently centrifuged at 11,000 *g* for 1 h at 4°C. The collected supernatants were submitted to protein concentration analyses using bovine serum albumin as standard (16). The pH that displayed greater solubility was considered as the relative solubility.

Solubility at different concentrations of NaCl was assessed using 5 mL of collagen solution, with a final concentration of 6 mg/mL, and a further 5 mL of 0, 2, 4, 6, 8, 10, and 12% (w/v) of a solution of 0.5 M acetic acid with NaCl yielding final concentrations of 0, 1, 2, 3, 4, 5, and 6% (w/v) of NaCl. These solutions were homogenized for 60 min and centrifuged at 11,000 *g* for 60 min at 4°C. The protein concentration was measured by the same method used for solubility in pH. The NaCl concentration that presented higher solubility was considered as the relative solubility.

The denaturation temperature (Td) was determined according to a previously published protocol with some modifications (17). A viscometer (Brookfield, Model DV-II, USA) was used, and a 6 mL collagen solution was added in a concentration of 1 mg/mL. The samples were heated from 20°C to 60°C in a 1°C/min range. The denaturation temperature was determined by fractional viscosity at 0.5, being considered as the denaturation temperature. The fractionated viscosity was determined by the formula: *Fractional viscosity* = [(ε_2_ *C* ^−1^) − (ε_3_ *C* ^−1^)] / [(ε_1_ *C* ^−1^) − (ε_3_ *C* ^−1^)], where, C: collagen concentration (mg/mL), ε1: viscosity at minimum temperature, ε2: viscosity at the measured temperature, and ε3: viscosity at maximum temperature. The physical-chemical analyses were performed in triplicate and results are reported as means±SD.

## Results

The centesimal composition of pirarucu skin tissue corresponded to the average of three regions: belly, loin, and tail (Table 1). All analyses were performed in triplicate, considering the average values to evaluate the content of lipids, proteins, and carbohydrates. The procedure used to extract the collagen obtained a yield of 27.74% of ASC from pirarucu skin tissue.

**Table 1:** Chemical composition (%) of pirarucu skin tissue *in natura*.

Region	Humidity	Ash	Lipids	Protein	Carbohydrates
Belly	59.55±0.26	0.20±0.01	17.72±0.19	21.92±0.71	0.61±0.07
Loin	53.64±0.67	0.16±0.01	21.03±0.73	22.20±1.17	2.97±0.12
Tail	47.64±0.26	0.20±0.02	19.27±0.66	31.43±2.73	1.46±0.10
Average	53.61	0.18	19.34	25.18	1.68

**Figure 2:**
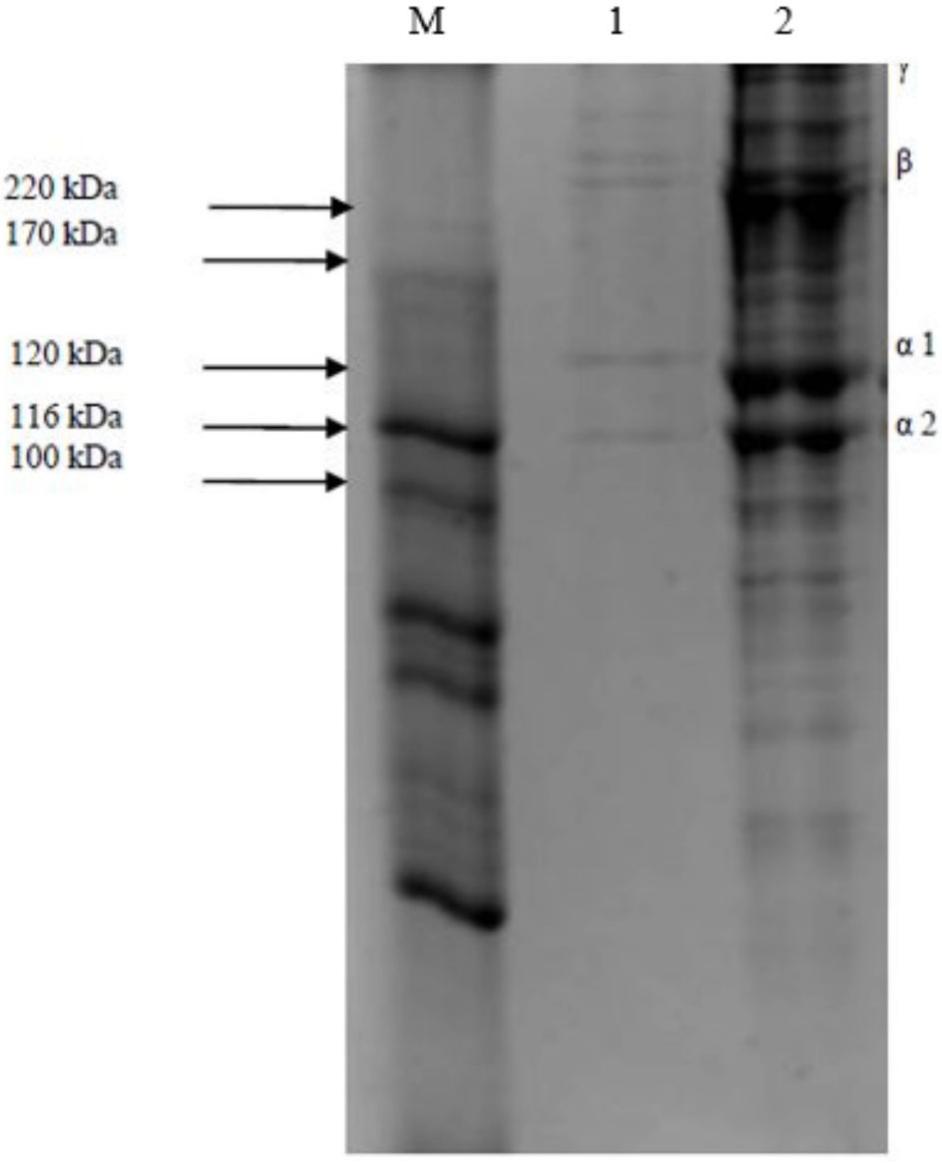
Polyacrylamide gel electrophoresis. M: High molecular weight marker; 1: commercial bovine standard type I collagen; 2: acid-soluble collagen (ASC) from pirarucu skin tissue. The marked presence of bands characteristic of type 1 collagen in the ASC obtained from pirarucu skin is observed; the α chains are shown below 200 kDa and the β chain above 200 kDa (29: doi: 10.1002/bip.360271105) as the γ chain, which is a typical feature of native collagen.

Figure 2 presents the electrophoretic pattern of high molecular weight marker (M) of type I collagen from commercial bovine standard and from the pirarucu skin tissue ASC. The sample presented two α-chains in proximal positions (α1 and α2), with a molecular mass between 100 and 120 kDa, and the β and γ subunits with a molecular mass above 200 kDa. A similar pattern was observed in bands of ASC from the sample and in type 1 collagen from bovine standard, characteristics of type I collagen reported by literature (18).

**Figure 3:**
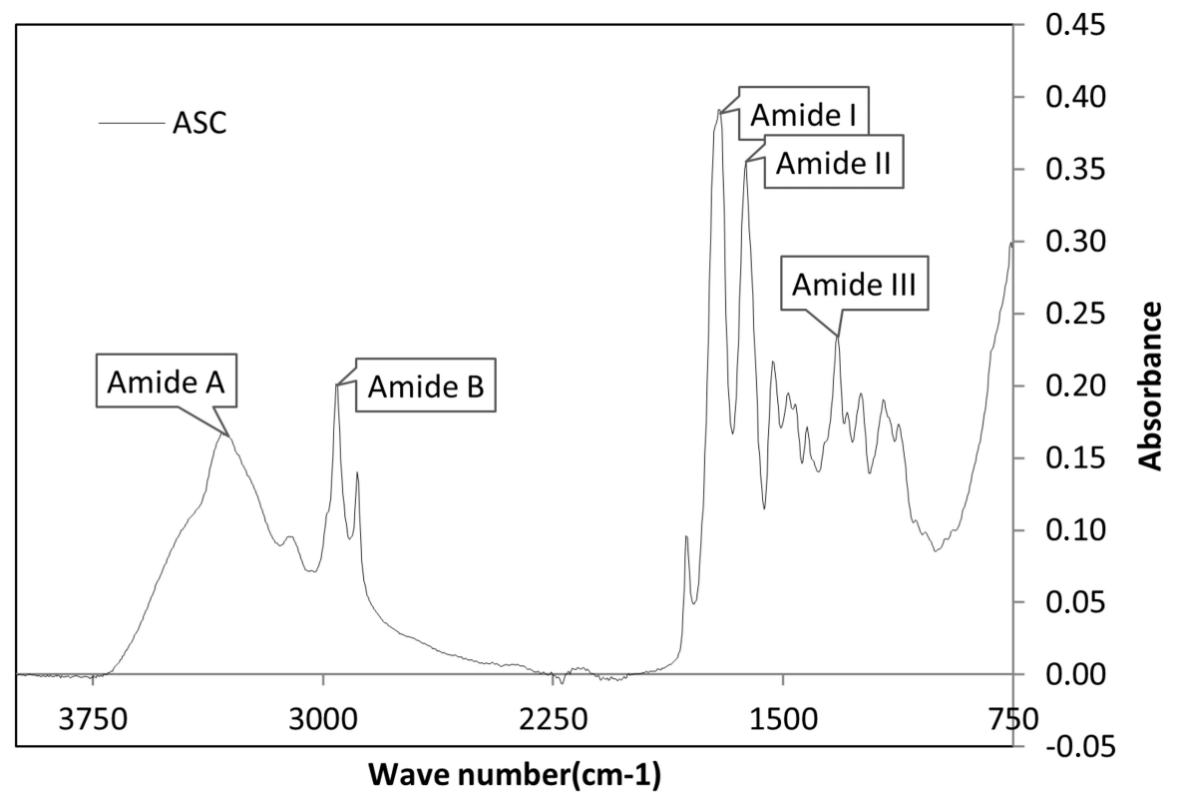
Fourier-transform infrared spectrophotometry analyses of the acid-soluble collagen (ASC) from pirarucu skin tissue. The spectrum shows the main functional groups of type 1 collagen.

The accuracy of the FTIR technique in the analysis of the chemical structure of samples helped to detect the vibrations of the functional groups present, where connections and groups of connections tend to vibrate at the moment of absorbing an infrared radiation at a specific wave number (cm^-1^) at the characteristic frequencies, and these spectra represent the molecular signature of the sample (19). In Figure 3 and Table 2, it is possible to observe the spectrum formed by the biomolecule (ASC) and its functional groups, with their respective meanings of vibration for each functional group present in the sample.

**Table 2:** Main spectral peaks of collagen from pirarucu skin tissue.

Wave number peak (cm^-1^)	Region
3307	Amide A. N-H vibration with hydrogen bands
2953	Amide B. N-H vibration
1704	Amide I. Vibration of carbonyl C=O
1621	Amide II. Vibration of carbonyl C=O, N-H bonds
1321	Amide III. C-H vibration

**Figure 4:**
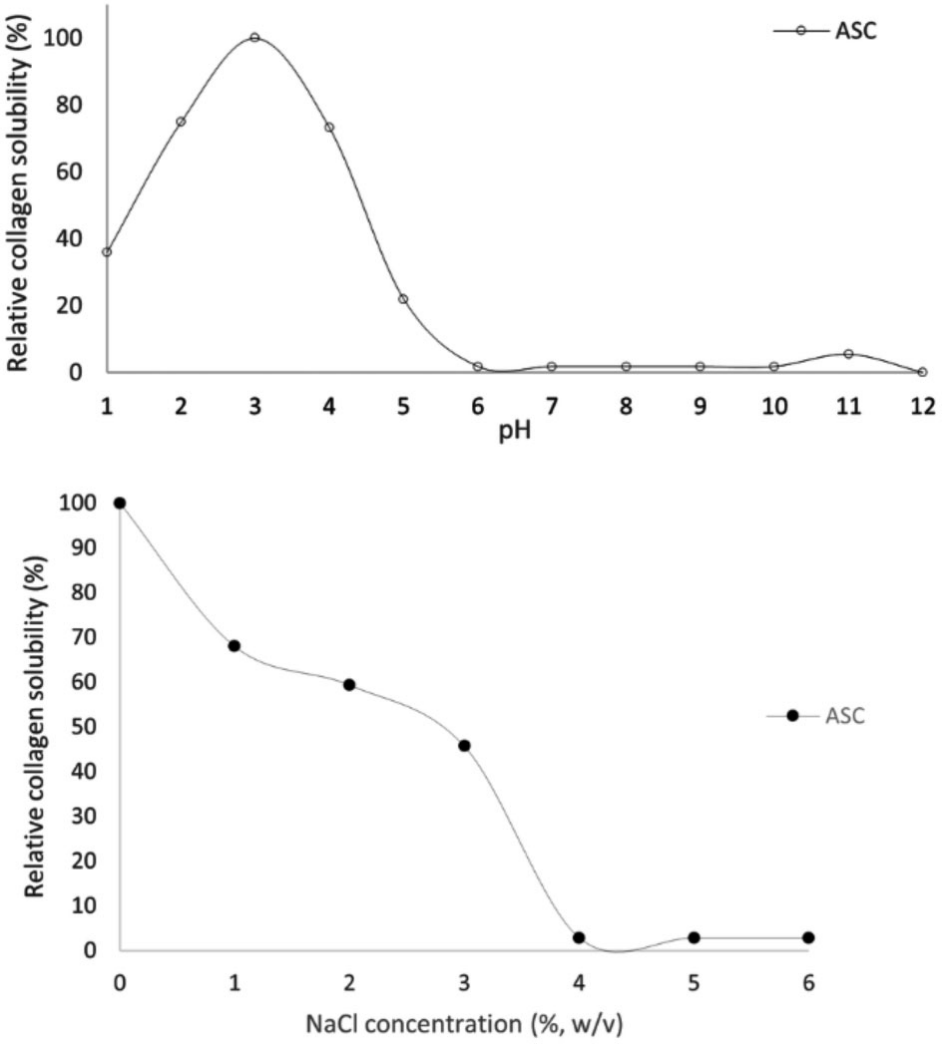
Relative solubility of acid-soluble collagen (ASC) from pirarucu skin tissue at different pH values and at different NaCl concentrations. There is a peak at pH 3 and a 50% loss of solubility at 3% NaCl.

The collagen behavior at different pH values and NaCl concentrations are presented in Figure 4. ASC presented good solubility between pH values 2 and 4, with a greater solubility at pH 3. However, at pH values below 2 and above 7, this solubility decreased by almost 50%.

Figure 4 presents the effect of different NaCl concentrations on ASC solubility. There was an evident decrease from 1% NaCl. At 3% NaCl concentration there was a loss in ASC solubility of 45.68%. At 4% NaCl, solubility was almost lost, indicating that collagen is not salt tolerant.

**Figure 5:**
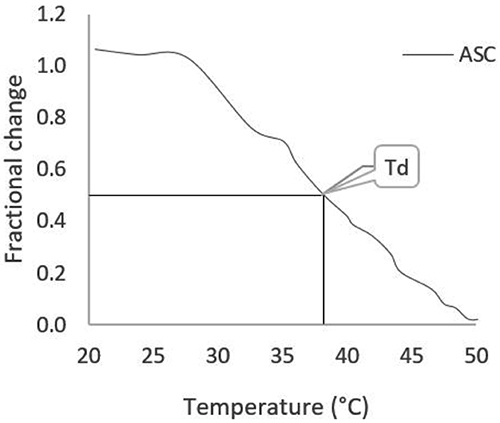
Denaturation temperature (Td) of acid-soluble collagen (ASC) from pirarucu skin tissue.

Denaturation temperature (Td) was considered the temperature when fractional viscosity was 0.5. Images were created using the Brookfield Engineering Labs program. Figure 5 presents the denaturation temperature of ASC obtained in the present study of 38.1°C.

## Discussion

The pirarucu skin tissue is a potential source of collagen due to its protein content. Humidity and protein values for pirarucu skin compared with other species such as tilapia skin are reported in other studies (20,21).

The lipid content of pirarucu skin influences collagen extraction, as it interferes in the penetration of solutions, thus the method to remove the skin from the fish is very important. The lipid content may be influenced by the size of the fish (22). In addition, nitrogenous components of lipids and the high content of unsaturated fatty acids give to the pirarucu skin its peculiar smell (23). Data presented by the authors (20,21) show a lower lipid content in tilapia skin compared to arapaima skin. The centesimal composition values of the pirarucu skin in this study were similar to those reported by other authors in terms of humidity and showed small differences in the values of proteins, lipids, and ash (24).

The extraction method produced approximately 27.74% of ASC from pirarucu skin tissue. This suggested that pirarucu skin tissue could be an alternative for collagen extraction. Studies using other fish species of tropical waters also observed a good yield of collagen using the same conditions and methods for extraction (4). The yield of ASC from pirarucu skin was similar to tropical fish: 22% in *Oreochromic niloticus* (25), 41.3% in *Cyprinus carpio* (7), and 51.4% in *Lateolabrax japonicus* (26).

The electrophoresis technique was used to identify the presence of ASC from the pirarucu skin based on the molecular weight. Figure 2 shows a similar pattern in the ASC and in the standard bovine type 1 collagen. The presence of more bands in the ASC may be due to the presence of other proteins still present in the sample or due to an incomplete purification process. However, the marked presence of bands characteristic of the ASC is evident, which is a typical feature of native collagen (18,27).

The absorption peaks from the FTIR technique at 3307 cm^-1^ and 2953 cm^-1^ are typical of amide A and amide B bands. Amide A was observed in the ASC sample at 3307 cm^-1^, indicating its association with existing N-H stretching vibrations of hydrogen bands (28).

The amide A bands obtained were similar to those in bovine standard collagen reported in other studies (3) with a value of 3296 cm^-1^. These free N-H stretching vibrations usually occur in the range of 3400 to 3440 cm^-1^ (28). However, when the N-H (amide) group of peptides is involved in hydrogenated bands, the position moves at lower frequencies around of 3300 cm^-1^ (Table 2 and Figure 3). This may indicate that many N-H groups of ASC were involved in the hydrogen bonds.

The amide B bands of collagen were observed at 2953 cm^-1^, being related to asymmetric stretching of CH_2_ (methylene). Similar values of 2933 cm^-1^ for bovine standard collagen were also found in other studies (3), which also showed similar differences in wavelength and amplitude of amide types A and B compared to bovine standard collagen. According to reference 3, the secondary structure of the triple helix can be different in some extensions, being likewise considered as collagen.

The bands of amide type I were frequent in the range of 1750 to 1600 cm^-1^, which may be associated with stretching vibrations of carbonyl group (C=O bands) along the polypeptide chain (29), being a sensitive marker of secondary structure of the peptide group, with its multiple structure occurring due the heterogeneity of C=O peptide groups in the triple helix, and influencing the spectral profile of bands of amide type I. The bands of amide type I of ASC were observed at 1704 cm^-1^, which was in line with values reported in literature. Types II and III amide bands in 1621 and 1321 cm^-1^ were the results of vibrations in N-H bonds and C-H stretches, respectively (29).

The absorption near 1633 cm^-1^ does not occur because collagen denatures (30), but the range of 1850-1628 cm^-1^ is considered a characteristic region in the absorption of carbon-oxygen double bond carbonyls, presenting a strong peak in this range due to the concentration of hydroxyproline as may be observed in Figure 3. The presence of the hydrogen bonds formed by water molecules and collagen molecules causes changes in FITR spectra (31). An example is the intensity of amide B peak (N-H stretches) presenting more changes in the amide II region (1621 cm^-1^). The stability of the collagen triple helix structure was determined by calculating the absorption radius between amide III and 1454 cm^-1^ (4), being the value near 1, which means that the molecular structure of ASC from pirarucu skin tissue remained stable.

The ASC from pirarucu skin tissue presented maximum solubility at pH 3. A decrease in collagen solubility may occur due to reaching the isoelectric point, where the solubility of protein is almost zero. At the same time, it is related to operational parameters and the integrity of its crosslinks, varying from species to species (25). The pH solubility values presented in this research are similar to those reported by other authors for other fish species (27,31,32).

The solubility of collagen declines with increasing salt percentage, starting at 1% NaCl. At 3%, ASC has a solubility loss of 45.68%, and near 4% NaCl, it loses almost all of its solubility. The increase in ionic forces causes a reduction in the solubility of protein due to the hydrophobic interactions between protein chains (33). Thus, the water increasingly competes with the salt ions, leading to protein precipitation (34). This result confirmed what has been reported by authors who blame the hydrophobic interactions as operational parameters for protein precipitation (27). The NaCl solubility values presented in this research are similar to those reported by other authors for other fish species (27,35).

The thermal stability of collagen is related to the habitat and body temperature (36) in the same way that Td of the same species may present small variations according to the method used to measure Td (37). Therefore, the collagen Td of fishes from temperate waters will always be lower than of fishes from tropical waters (38). The application of high temperatures to collagen causes the breakdown of hydrogen bonds and intermolecular bonds and changes its triple helix structure. In general, the thermal stability of collagen is assessed by its denaturation temperature (Td) as well as its shrinkage temperature (Ts) (39).

The Td of the ASC from pirarucu skin tissue was 38.1°C. The Td of collagen is defined as the temperature at which the viscosity is partially changed. Td values of collagen from porcine was 37°C (26), 32°C for collagen from *Oreochromic niloticus* (25), 28°C for collagen from *Cyprinus carpio* (40), and 25.6°C for collagen from *Scomber japonicus* (26).

In conclusion, it was possible to obtain collagen from pirarucu skin tissue with similar physical and chemical characteristics of commercial type I bovine collagen. The extraction process used could be a viable alternative for obtaining collagen from Amazonian fish species. However, we recommend adjusting operating parameters to optimize the extraction process. Further studies are necessary to fully characterize the obtained collagen.
